# Visual complaints in pregnancy: (pre)eclampsia as a chameleon

**DOI:** 10.3205/oc000208

**Published:** 2022-11-21

**Authors:** Johanna Wiedemann, Lebriz Altay, Antje Neugebauer, Tim Krohne, Claus Cursiefen

**Affiliations:** 1 Department of Ophthalmology, Faculty of Medicine and University Hospital of Cologne, Germany

**Keywords:** PRES, DIC, diabetes type I, pregnancy, (pre)eclampsia

## Abstract

**Objective:**

The visual system often is affected in patients with preeclampsia and even more in cases of eclampsia, a life-threatening pregnancy complication. Symptoms include blurred vision and deterioration of visual acuity. Pregnancy can also affect pre-existing conditions, such as diabetic retinopathy. In this case series, we describe three patients with the same underlying condition, i.e. (pre)eclampsia who experienced acute visual disturbance whereas the final diagnosis was different: disseminated intravascular coagulopathy (DIC), posterior reversible encephalopathy syndrome (PRES), and diabetic retinopathy.

**Methods and results:**

All patients underwent a thorough slit lamp examination and ocular coherence tomography (OCT). All patients presented with acute impaired vision and subretinal fluid and-/or fibrin.

**Conclusions:**

These cases highlight the importance of early involvement of ophthalmologists when pregnant women complain about visual disorders.

## Introduction

Preeclampsia is an obstetric complication occurring in 3–5% of pregnancies and presents as a symptomatic triad of hypertension, proteinuria and edema, whereas eclampsia adds convulsions to this triad [1]. HELPP syndrome, which is an acronym for hemolysis, elevated liver enzymes and a low platelet count is often a complication of the two aforementioned conditions. The maternal (3%) and infantile (24%) mortality rates in patients developing preeclampsia are high, in patients with eclampsia and HELPP even higher [2], [3], [4]. 

Crucial for improvement in all cases is the treatment of the underlying disease, i.e. (pre)eclampsia, by either treating conservatively in mild conditions or if severe starting with birthing the child and managing symptoms. Pregnancy state, severity of (pre)eclampsia and child condition all play an important role in therapy decisions. Conservative treatment requires an intensive care setting with a continuous monitoring of vital parameters and laboratory values. It aims mainly at symptom control by administering antihypertensive and if necessary anticonvulsive medication [5].

## Case description

### Case 1: Disseminated intravascular coagulopathy (DIC) 

**Figure 1:**
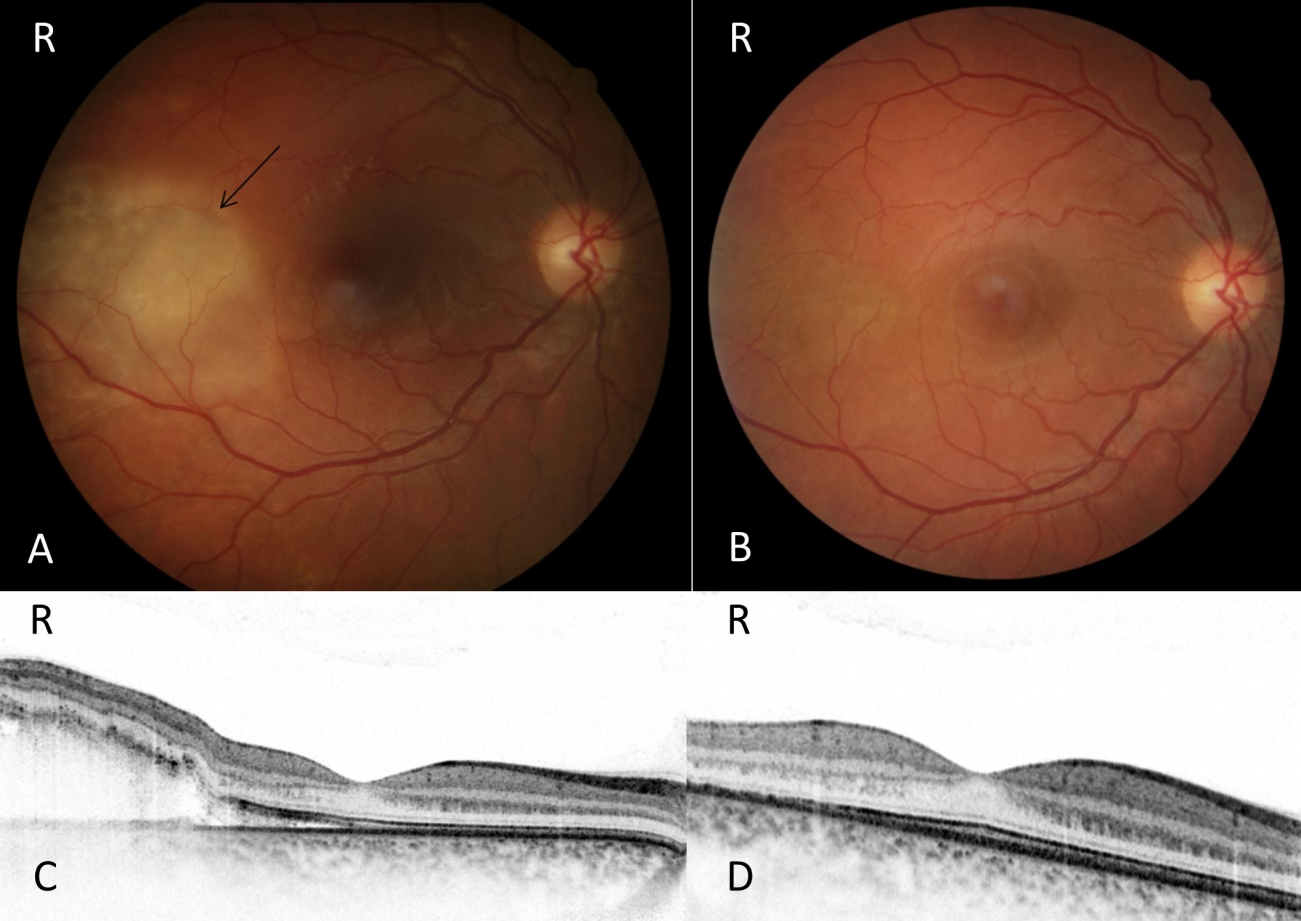
Fundus changes in DIC. A: Funduscopic image of patient 1 with DIC at baseline showing a whitish lesion (arrow). B: Funduscopic image at follow up (t=14 days) showing a normal background with a complete regression of the previous seen whitish lesion. C: OCT at baseline showing subretinal material. D: OCT at follow up (t=14 days) showing a complete resolution of the subretinal material.

A 31-year-old patient was referred to our hospital two days after her caesarean section (C-section) in the 38^th^ week of pregnancy. The patient was treated in our obstetrics department because she was suffering from eclampsia and HELPP syndrome. Due to visual complaints, she was sent for a co-assessment by the ophthalmology department. Her symptoms included blurred vision and disturbed color perception exclusively in the right eye. Best corrected visual acuity (BCVA) at baseline was 20/20 in both eyes. Eye motility and pupillary reaction were normal. She had a normal slit lamp examination. She perceived double vision in right gaze but showed ocular alignment in primary position. Funduscopic findings included a large whitish retinal zone in the temporal, inferior retinal quadrant, without any signs of bleeding (Figure 1 A, B (Fig. 1)). Ocular coherence tomography (OCT) showed unilateral subretinal fluid (SRF) and fibrin accumulation corresponding to the whitish lesion on the funduscopic image. Fluorescein angiography was not performed due to ongoing lactation. In the OCT angiography, no (secondary) choroidal neovascularization could be detected. Funduscopically no signs of ischaemia or insufficient perfusion could be detected. The patient was closely monitored in our clinic without any intervention. Four days after C-section the subretinal material started to regress and had completely resolved 2 weeks after the initial presentation (Figure 1 C, D (Fig. 1)). 

Approximately 40% of women with preeclampsia/eclampsia report visual impairment. In severe cases of preeclampsia/eclampsia serous retinal detachments have been reported due to choroidal perfusion impairment [6]. In this case, the cause of perfusion impairment may be related to disseminated intravascular coagulation (DIC).

Per definition, DIC is not a disease in itself but an acquired clinicobiological syndrome characterized by sometimes widespread vascular endothelial damage, activation of coagulation and thrombus formation in small vessels, and the related organ dysfunction [7], [8]. DIC is provoked by several underlying disorders including pregnancy complicated by eclampsia [9]. In cases of (pre)eclampsia associated DIC, the maternal mortality rate is over 25% and perinatal child mortality around 47% [7]. 

A battery of laboratory tests (prothrombin time, partial thromboplastin time, thrombin time, and plasma fibrinogen) can be used in the diagnosis. However, no single test in isolation is sensitive and specific for diagnosis. The diagnosis is a diagnosis by exclusion. In our patient, the diagnosis of DIC was established due to the patient’s history, the ophthalmologic findings, and the laboratory tests. 

Differential diagnosis of DIC includes other causes of consumptive coagulopathies, such as trauma or major surgery. In patients with severe liver disease with reduced production of coagulation factors and inhibitors or in thrombocytopenia secondary to splenic sequestration a similar clinical picture may be seen [7], [8]. 

Several obstetric disorders such as placental abruption, amniotic fluid embolism, sepsis syndrome, and acute fatty liver of pregnancy are associated with DIC. However, none of these conditions occurred in our patient [9]. 

### Case 2: Posterior reversible encephalopathy syndrome (PRES) 

**Figure 2:**
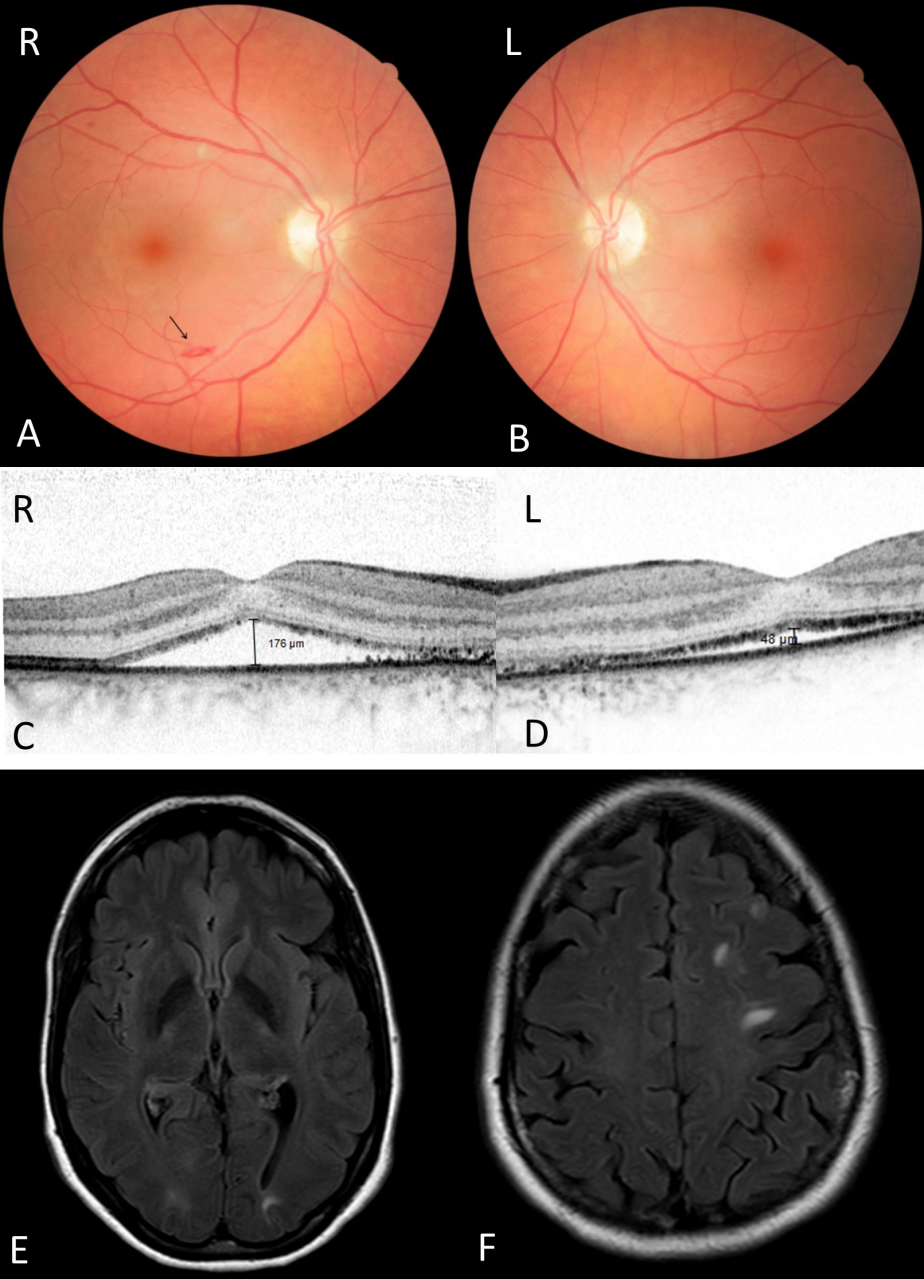
Retinal and radiological changes in PRES. A, B: Funduscopic image of patient 2 with PRES at baseline showing a small bleeding (arrow) in the right eye. C, D: OCT of patient 2 with PRES at baseline showing bilateral subretinal fluid. E: MRI image of patient 2 with PRES showing hyperintensity in the temporo-parietal region. F: MRI image showing hyperintensity in the occipital region.

A 36-year-old patient was referred to our hospital one week after an emergency C-section in the 24^th^ week of pregnancy because of binocular diplopia and blurred vision. The C-section was necessary due to the development of preeclampsia with HELPP syndrome. Both eyes had a BCVA of 20/25, no strabismus in primary position, normal motility and a normal slit lamp examination. Funduscopy of the right eye revealed a punctual retinal bleeding near the inferior branch of the retinal artery as well as a cotton wool spot near the superior branch of the retinal artery (Figure 2 A, B (Fig. 2)). In baseline OCT, SRF was detected (Figure 2 C, D (Fig. 2)). As the patient also complained of headache beginning two weeks before delivery, a magnetic resonance imaging (MRI) was effectuated immediately after the C-section. 

Symmetrical white matter abnormalities suggestive of edema can be seen in the computer tomography (CT) and MRI scans, mostly but not exclusively in the posterior parieto-occipital regions (Figure 2 E, F (Fig. 2)). 

The diagnosis of posterior reversible encephalopathy syndrome (PRES) was based on the combination of symptoms and the neuro-radiological findings.

PRES is a rare neurological disorder with still not completely explained pathophysiology. Like DIC, PRES it is not a disease in itself but an entity that can occur in different contexts such as renal diseases or (pre)eclampsia.

PRES is mostly associated with hypertension and endothelial injury. Vasoconstriction resulting in vasogenic and cytotoxic edema is suspected of being responsible for the clinical symptoms as well as the radiological image [10]. 

Differential diagnosis to explain the visual symptoms in this patient includes DIC, functional postpartal visual disturbance, pseudotumor cerebri, and bilateral retinal detachment due to severe preeclampsia. All these differential diagnoses could be excluded either by the funduscopic or radiological findings.

In the follow-up OCT examination, the SRF was decreasing. In the follow-up MRI 3 months later, the parietal-temporal cerebral edema, correlating with PRES, was completely resolved. 

The treatment of PRES is symptomatic and focused on the underlying condition with an overall favorable prognosis. Clinical symptoms as well as imaging lesions are reversible in most patients. However, neurological sequelae including long-term epilepsy may persist in individual cases [10]. 

### Case 3: Type 1 diabetes 

A 26-year-old patient presented with blurry vision in her 20^th^ week of pregnancy. Visual acuity was 20/200 in her right eye and 20/32 in her left eye. She had diabetes type I which was fairly dysregulated with a hemoglobin A1c (HbA1c) of 7.0% at baseline. The patient had no ophthalmological treatment before and during the pregnancy and now presented with proliferative diabetic retinopathy (PDR) with bilateral retinal neovascularization which was treated with bilateral panretinal laser coagulation. OCT showed bilateral diabetic macular edema, however an intravitreal application of a vascular endothelial growth factor (VEGF) antagonist drug during pregnancy was not consented by the patient.

In the further course, visual acuity decreased within 3 months to hand movements in both eyes with increasing macular edema and papillary swelling. Additional symptoms of preeclampsia and severe diabetic nephropathy occurred in the 28^th^ week so we referred the patient to our obstetric department where a C-section was performed. 

**Figure 3:**
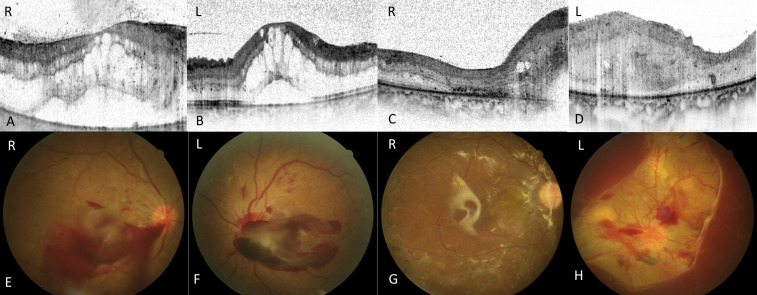
DR in preeclampsia. A, B: OCT of patient 3 with DM type I at baseline showing bilateral cystic macular oedema (CME). C, D: OCT at follow up (t= 6 month) showing a regressive CME in the right eye and fibrovascular material in the left eye. E, F: Funduscopic image of patient 3 with DM type I at baseline showing bilateral preretinal hemorrhages. G: Funduscopic image at follow up (t=6 month) showing a regression of preretinal hemorrhage. H: Funduscopic image at follow up (t=6 month) showing an aggravation of the preretinal hemorrhage.

Two weeks after the C-section, both eyes still showed massive proliferations on the posterior pole, macular edema, tractive retinal membranes and bleeding (Figure 3 (Fig. 3)). 

Both eyes underwent pars plana vitrectomy in a short period, with removal of tractive membranes, peeling of the inner limiting membrane, additional panretinal laser treatment and injection of intravitreal steroids (for the right eye) and anti-VEGF substances (both eyes). 

A stable retinal condition was achieved 1.5 years after the initial complaint with a visual acuity of 20/400 in both eyes. 

The prevalence of diabetes in pregnant women is constantly increasing, it is a leading cause of blindness in women during their childbearing years, and pregnancy increases the short-term risk of diabetic retinopathy progression occurring in 77.5% up to one year postpartum [11], [12], [13]. In cases of preexisting diabetes, usually type I, the condition can also be affected during pregnancy [14]. Risk factors for progression include baseline level of retinopathy, level of glycemic control and hypertension [13]. 

The underlying disease therefore is responsible for the potential exacerbation as well as for an increased risk of pregnancy complications such as (pre)eclampsia as occurred in our patient [15]. 

Resulting complications such as nephropathy, as occurred in this patient, are associated with an increased risk of nephrotic syndrome, preterm delivery, fetal growth restriction, and perinatal mortality. Presence of retinopathy and poor glycemic control also increase the risk of preeclampsia. The pregnancy itself (first or subsequent) is not a long-term risk factor for developing any retinopathy, proliferative retinopathy, or neuropathy.

Good glycemic control, normotension, no nephropathy as well as no pre-proliferative/proliferative changes of diabetic retinopathy and no signs of macroangiopathies are good prognostic factors as regards the progression of vascular complications during pregnancy. Women with diabetes should be evaluated before pregnancy for microangiopathies, treated and followed closely during pregnancy by obstetricians, internists (diabetologist, cardiologist, nephrologist) and ophthalmologists. Our patient is now closely followed by internists and ophthalmologists.

## Discussion

Visual perturbances occur in different shapes and forms. However it is essential to be properly looked at when expressed. Ocular symptoms are very frequent in patients with eclampsia (up to a 100%) and less in cases of preeclampsia (22%). Fundus changes occur in around 48.7% of affected patients [16].

Most important for the improvement of the situation is the detection of the underlying pathology, as it will guide treatment. The (pre)eclampsia was treated by C-section, according to clinical standards, and in all three patients without further complications. All patients received regular ophthalmological follow-ups and showed improvement or stabilization of their condition.

Further differential diagnosis includes central serous chorioretinopathy and hypertensive retinopathy.

## Conclusion

Visual disturbances during pregnancy may have different reasons and the underlying possible systemic reason should be evaluated carefully. In cases of visual disturbances during pregnancy a full ophthalmologic check-up should be performed including fundoscopy in mydriasis in order to detect possibly treatable conditions. Sometimes ophthalmic changes preceed systemic ones, and thus the ophthalmologist should initiate further examinations by other subspecialities.

## Notes

### Ethics statement

All patients gave written informed consent. This report does not contain any personal information that could lead to the identification of the patient. According to national medical regulations in retrospective single center clinical studies Ethics Committee of the University of Cologne ruled that approval was not required. All tenets of the declaration of Helsinki were regarded. The study was in accordance with the tenets of the Declaration of Helsinki and the Medical Research Involving Human Subjects Act (WMO).

### Competing interests

The authors declare that they have no competing interests.
